# Metabolic Peculiarities of *Paracoccidioides brasiliensis* Dimorphism as Demonstrated by iTRAQ Labeling Proteomics

**DOI:** 10.3389/fmicb.2019.00555

**Published:** 2019-03-20

**Authors:** Danielle Silva Araújo, Maristela Pereira, Igor Godinho Portis, Agenor de Castro Moreira dos Santos Junior, Wagner Fontes, Marcelo Valle de Sousa, Leandro do Prado Assunção, Lilian Cristiane Baeza, Alexandre Mello Bailão, Carlos André Ornelas Ricart, Matthias Brock, Célia Maria de Almeida Soares

**Affiliations:** ^1^Laboratório de Biologia Molecular, Instituto de Ciências Biológicas, Universidade Federal de Goiás, Goiânia, Brazil; ^2^Laboratório de Bioquímica e Química de Proteínas, Instituto de Ciências Biológicas, Universidade de Brasília, Brasília, Brazil; ^3^Faculdade Unida de Campinas, Goiânia, Brazil; ^4^Fungal Biology and Genetics Group, University of Nottingham, Nottingham, United Kingdom

**Keywords:** iTRAQ, proteomics, neglected disease, paracoccidioidomycosis, dimorphism

## Abstract

Paracoccidioidomycosis (PCM), a systemic mycosis with a high incidence in Latin America, is caused by thermodimorphic fungi of the *Paracoccidioides* genus. The contact with host occurs by the inhalation of conidia or mycelial propagules which once reaching the pulmonary alveoli differentiate into yeast cells. This transition process is vital in the pathogenesis of PCM allowing the fungus survival in the host. Thus, the present work performed a comparative proteome analysis of mycelia, mycelia-to-yeast transition, and yeast cells of *Paracoccidioides brasiliensis*. For that, tryptic peptides were labeled with iTRAQ and identified by LC–MS/MS and computational data analysis, which allowed the identification of 312 proteins differentially expressed in different morphological stages. Data showed that *P. brasiliensis* yeast cells preferentially employ aerobic beta-oxidation and the tricarboxylic acid cycle accompanied by oxidative phosphorylation for ATP production, in comparison to mycelia and the transition from mycelia-to-yeast cells. Furthermore, yeast cells show a metabolic reprogramming in amino acid metabolism and in the induction of virulence determinants and heat shock proteins allowing adaptation to environmental conditions during the increase of the temperature. In opposite of that, the alcoholic fermentation found to *P. lutzii*, at least under laboratory conditions, is strongly favored in mycelium compared to yeast cells. Thereby, the data strongly support substantial metabolic differences among members of the *Paracoccidioides* complex, when comparing the saprobiotic mycelia and the yeast parasitic phases.

## Introduction

Dimorphic fungi of the *Paracoccidioides* genus cause a systemic mycosis called Paracoccidioidomycosis (PCM) (Brummer et al., 1993; Marques-da-Silva et al., 2012). The genus *Paracoccidioides* was previously described to comprise a single species but more recent classifications divided the genus in five species: *P. brasiliensis*, *P. americana*, *P. restrepiensis*, *P. venezuelensis*, and *P. lutzii* (Teixeira et al., 2009; Turissini et al., 2017).

Under free environmental conditions or during *in vitro* cultivation at 22–25°C, members of this genus develop hyphae and form a mycelium. However, in host tissue or when cultivated at 36°C, these organisms display a yeast cell morphology (Brummer et al., 1993). The mycelium generally decomposes organic matter in soil that is necessary for environmental survival. Moreover, mycelia can respond to different environmental conditions such as changes in temperature and humidity and competition with other microorganisms (Barrozo et al., 2009). Human infection initiates through the inhalation of conidia or hyphal fragments, which reach the pulmonary alveoli and transit to the yeast form in response to the increased temperature in the body (Smith and Kauffman, 2012; Buccheri et al., 2016).

The transition from mycelium into the yeast phase is essential for members of the *Paracoccidioides* complex to establish the disease, since strains which do not differentiate into yeast cells are avirulent (Nemecek, 2006). Therefore, identification of genes and proteins involved in the mycelia-to-yeast transition has been subject of interest, due to the fact that pathogenicity is linked to the dimorphism (Rooney and Klein, 2002). In previous studies, the transcriptome of *Paracoccidioides lutzii* mycelium and yeast cells have been investigated and provided insights into metabolism in the different fungal phases (Felipe, 2005). The transcription profile of mycelium suggested the shunting of pyruvate into aerobic metabolism, whereas in yeast cells pyruvate produced by glycolysis undergoes a fermentative metabolism (Felipe, 2005).

Transcriptomic analysis of *P. brasiliensis* derived from mycelium-to-yeast transition was performed by monitoring the expression of 4,692 genes at several time points of the transition period by using microarray analyses (Nunes et al., 2005). The data revealed 2,583 genes differentially expressed during transition, which were involved in cellular processes such as cell wall metabolism, signal transduction, and oxidative stress response. The transcriptome analysis of early morphogenesis in *P. lutzii* mycelium undergoing transition to yeast cells, performed at our laboratory, revealed 179 genes with positive regulation at the early transition process when compared to mycelia (Bastos et al., 2007). Of special note, genes encoding proteins of fungal cell wall and membrane remodeling were positively regulated during mycelium-to-yeast transition. In this class were included those genes related to the cell wall carbohydrates biosynthesis and degradation, transporters of the precursors for the synthesis of those molecules, and enzymes related to protein glycosylation and to the synthesis of membrane lipids. Notably, 34 expressed sequenced tags (ESTS) were significantly induced, whose cognate proteins were supposed to work in cell wall/membrane remodeling in the 22 initial hours of the transition from mycelia-to-yeast cells. The data strongly suggest that *P. lutzii* prioritizes the membrane and cell wall remodeling in the initial stages of the transition from mycelium to yeast cells (Bastos et al., 2007).

In a pioneering quantitative 2-D electrophoresis-(2-DE) based proteomic study of the morphological phases of *P. lutzii*, Rezende et al. (2011), at our laboratory, detected changes in the relative abundance of proteins in mycelia, transition from mycelia-to-yeast, and yeast cells (Rezende et al., 2011). This resulted in detailed 2-DE reference maps of proteins in the mycelia, yeast cells, and transition from mycelia-to-yeast and revealed a global reorganization of the *P. lutzii* metabolism during transition from mycelia-to-yeast cells (Rezende et al., 2011). A major change was detected in the accumulation of glycolytic enzymes and of alcohol dehydrogenase at 22 h after the mycelium-to-yeast transition, consistent with transcriptional studies that have detected a change toward anaerobic metabolism in the yeast phase of *P. lutzii* (Felipe, 2005).

Metabolic differences are described in members of the *Paracoccidioide*s genus that could account to their adaptation to the host (Pigosso et al., 2013; Baeza et al., 2017; Lacerda Pigosso et al., 2017). Previous work from our group evidenced that when yeast cells have glucose as the carbon source, proteins related to glycolysis/gluconeogenesis and to alcohol fermentation were more accumulated in *P. lutzii* when compared to other members of the genus. Moreover, the oxidative stress response was more evidenced in *P. lutzii*, *P. americana*, and *P. restrepiensis* (Pigosso et al., 2013). When yeast cells were grown in acetate as a carbon source, the metabolic profile depicted gluconeogenesis and catabolism of amino acids induced at similar rates among members of the *Paracoccidioides* genus. For *P. lutzii* and *P. restrepiensis*, β-oxidation, TCA, and glyoxylate cycles were increased in comparison to *P. brasiliensis* and *P. americana* (Baeza et al., 2017). The data cited above reinforce the proposition of metabolic differences in members of the *Paracoccidioide*s genus.

Proteomics can offer unique large-scale data on cellular differentiation, as we had previously described (Rezende et al., 2011). Integration of proteomic data here demonstrated that in the yeast phase of *P. brasiliensis*, a metabolic reprogramming occurs in pathways such as beta-oxidation, methylcitrate cycle, and amino acid metabolism. Furthermore, induction of virulence factors and heat shock proteins (HSPs) that potentially allow the fungus to adapt to new environmental conditions were observed. The alcoholic fermentation appears more abundant in mycelium of *P. brasiliensis* compared to the yeast form. Proteins related to β-1,3-glucan synthesis required for the mycelium to construct a cell wall enriched in β-glucan polymers were annotated. In addition, superoxide dismutase (SODs) and thioredoxins (Trx) important for the mycelial phase to increase protection against oxidative stress were also found to be differentially regulated.

## Materials and Methods

### Microorganisms and Culture Conditions

*Paracoccidioides brasiliensis*, *Pb*18, was used in this study. The mycelium and yeast phases were maintained *in vitro* in solid BHI medium with 4% (w/v) glucose at 22 and 36°C for 15 and 7 days, respectively. The mycelium and yeast cells were transferred to liquid BHI with glucose, 4% (w/v) at 22 and 36°C under constant agitation (150 rpm) for 72 h. Mycelium-to-yeast transition was also performed in liquid BHI medium. The fungus was initially incubated at 22°C for 18 h and after this period, the temperature was shifted to 36°C, for 22 h. The whole procedure of sample preparation and analysis is schematically depicted in Supplementary Figure S1.

### Extraction of Proteins

Mycelia, mycelia in transition to yeast, and yeast cells were collected by centrifugation at 1,200 ×*g* for 10 min at 4°C, washed three times with PBS, and resuspended in lysis buffer (8 M Urea, 75 mM NaCl, 50 mM Tris, pH 8.2, 50 mM β-glycerol phosphate, 1 mM sodium orthovanadate, 10 mM sodium pyrophosphate, 1 mM PMSF). After addition of glass beads, cells were mechanically lysed by vigorous shaking using a mini beadbeater (BioSpec Cat. No. 607EUR). For removing cell debris, centrifugation at 10,000 ×*g* for 15 min at 4°C was performed. Proteins contents were estimated using the Qubit protein assay kit (Thermo Scientific, Bremen, Germany) and confirmed on 12% SDS–PAGE gel. The supernatants were stored at -80°C.

### Sample Preparation for LC–MS/MS Analysis

Before performing protein digestion, an acetone precipitation step was performed on the cell extracts (Lin et al., 2015). A total of 150 μg of protein sample was added of ice-cold acetone at volume ratio 1:5 and incubated for 16 h at -20°C. Subsequently, the samples were centrifuged at 1,500 ×*g*, for 5 min, followed by supernatant removal. The pellet was resuspended in lysis buffer [8 M urea, 0.05 M triethylammonium bicarbonate buffer (TEAB), pH 7.9].

A trypsin digestion was performed on the acetone-precipitated and resuspended proteins. The sample in lysis buffer was maintained at 4°C and sonicated for 60 s. Subsequently, a reduction step was performed for 25 min at 55°C by the addition of 0.005 M dithiothreitol. Following, iodoacetamide (0.014 M) was added and the samples were held for 40 min at room temperature, in the dark. Soon after, DTT was added to the sample at a final concentration of 0.005 M. To this sample 0.025 M TEAB, pH 7.9, 0.001 M CaCl_2_, was added at a volume ratio of 1:5. Digestion was then performed by addition of trypsin (Promega) at the enzyme:substrate ratio of 1:50, with incubation at 37°C for 12 h. The peptide samples were acidified with 0.1% TFA (v/v). Then, 50 μg of the sample was desalted in a StageTip on the low-binding P-200 tip with a C18 disc, and the rest of the sample was stored at -80°C. The eluate resulting from desalting was collected and dried in vacuum.

### iTRAQ Labeling

For iTRAQ labeling of protein samples of biological triplicates from each cultivation condition, the manufacturer’s specifications were followed with some modifications. A total of 50 μg desalted and dried peptide was resuspended in 17 μL of 300 mM TEAB. Then iTRAQ reagent, which was resuspended in 70 μL of ethanol, was added. The solution was incubated at room temperature for 2 h, followed by mixing all the labeled samples in equal proportion (mycelium 114; transition 116; and yeast 115). Samples were desalted with a StageTip on the low-binding P-200 tip with C18 matrix, and dried under vacuum.

### Data Acquisition by LC/MS–MS

The tryptic peptides were separated using a capillary column chromatography system (nano-UHPLC Dionex Ultimate 3000) coupled to a hybrid ion Trap-Orbitrap Mass Spectrometer, Orbitrap Elite^TM^ (Thermo Scientific). The first chromatography was carried out on a pre-column with internal diameter of 100 μm × 200 mm in length, packed in-house with silica spherical particles coated with C18 Reprosil-Pur of 5 μm with 120 Å pores (Dr. Maisch GmbH, Ammerbuch, Germany). The second chromatography was carried out using an analytical column of internal diameter of 75 μm and 350 mm in length, also packed in-house with C18 Reprosil of particles 3 μm with 120 Å pores (Dr. Maisch GmbH, Ammerbuch, Germany). The gradient for sample elution was 100% phase A (0.1% formic acid) to 26% phase B (0.1% formic acid, 95% ACN) for 180 min; 26–100% phase B for 5 min; and 100% B phase for 8 min (a total of 193 min at 200 nL/min). After each run, the column was washed with 90% B-phase and re-equilibrated with phase A.

The spectra were acquired in positive mode by applying data-dependent automatic MS scan and acquisition of mass spectra in tandem (MS/MS). MS/MS of the 15 most intense ions in the LTQ followed all MS scans in the orbitrap (mass amplitude: *m*/*z* 350–1,800 and resolution: 120,000). Fragmentation in LTQ occurred by high-energy collision-induced dissociation; selected ion sequences were dynamically deleted every 15 s. The search and identification of proteins used Proteome Discoverer v.1.3 beta software (Thermo Scientific-OPTON-30795) with Mascot algorithm v.2.3 against *P. brasiliensis* database installed on the lab server and generated using the Database on Demand tool containing the proteins found in UniProt/SWISS-PROT, UniProt/TrEMBL. The searches were made according to the parameters: MS precision of 10 ppm, MS/MS of 0.05 Da, until two cleavage sites lost; carbamidomethylation of cysteines, oxidation of methionine, and N-terminal acetylation of proteins as variable modifications. The number of proteins, the group of proteins, and the number of peptides were filtered with a false positive detection rate (FDR) of less than 1%; a minimum of two peptides per protein were accepted for identification with Proteome Discoverer. The Protein Center software (Thermo Scientific) was used to interpret the identified proteins.

### Statistical Analysis

The differences in protein expression among the three conditions were evaluated using the ANOVA and Tukey’s test; the latter was applied after statistically significant results were obtained by ANOVA and was used to compare the differences among the means in analyzed groups. Statistical analyses were performed using R software^1^. A *p*-value ≤ 0.05 was considered statistically significant. Only proteins detected in at least two replicates were evaluated.

### Bioinformatics Analysis

For the identified proteins, we did a search using the Blast2GO^2^. The annotations of the identified proteins were performed using BLASTP with a BLAST Expect value of 10^-3^ and a maximum number of 30 hits in a non-redundant protein sequence database. After these analyses, the mapping and annotation steps were performed (Conesa et al., 2005). The identified proteins were classified into functional categories based on the MIPS Functional categories database (Funcat 2.0)^3^, available in pedant^4^. The heat maps were generated using the package *heatmap.plus* of software R (R Core Team, 2018). The Venn diagram were generated using venny integrative tool available in http://bioinfogp.cnb.csic.es/tools/venny/index.html (Bardou et al., 2014).

### Determination of Ethanol Concentrations in Fungal Lysates

A total of 2 g of yeast cells, mycelium, and mycelium-to-yeast cells in transition were used to perform the assay. Briefly, the cells were lysed by the use of glass beads and mini beadbeater equipment in four cycles of 30 s, keeping the samples on ice in the interval of each cycle. The cell lysates were centrifuged at 10,000 ×*g* for 15 min at 4°C for obtaining the supernatant used for the enzymatic assay. The concentration of ethanol was quantified by using an enzymatic detection kit (UV-test for ethanol, RBiopharm, Darmstadt, Germany). Briefly, ethanol is oxidized to acetaldehyde in the presence of the enzyme alcohol dehydrogenase. Subsequently, acetaldehyde is oxidized quantitatively to acetic acid in the presence of aldehyde dehydrogenase and NAD^+^, releasing NADH, which is determined at absorbance at 340 nm. Concentrations of ethanol were determined in biological triplicates.

### Determination of Reduced Thiol Level

Concentrations of thiol were determined in biological triplicates. Briefly, a total of 2 g of mycelium, yeast cells, and mycelium-to-yeast cells in transition were used to perform the assay. The cells were disrupted in lysis buffer (50 mM Tris–Cl, 150 mM NaCl, 50 mM ethylenediamine tetraacetic acid [EDTA], pH 7.2), by adding glass beads in a mini beadbeater equipment in four cycles of 30 s; the samples were kept on ice in the interval of each cycle. The cell lysates were centrifuged at 10,000 ×*g* for 15 min at 4°C for obtaining the supernatant which was used for enzymatic assay. Thereafter, 100 μL of supernatant was combined with 100 μL of 500 mM sodium phosphate buffer, pH 7.5, and transferred into a microtiter well, followed by adding 20 μL of 1 mM 5,50-dithio-*bis*-(2-nitrobenzoic acid) (DTNB). Absorbance was determined at 412 nm using a plate reader. The reaction principle is based on the fact that Trx is reduced to dithiol T(SH)2 by Trx reductase (TR), in the Trx system. The enzyme inhibition promotes decrease in the amount of total reduced thiol (De Souza Bonfim-Mendonça et al., 2014). Free thiol levels were determined using the Ellman’s reagent, DTNB (Sigma–Aldrich, Co.).

### Real-Time RT-PCR

RNAs which were obtained by using Trizol were treated with DNase (RQ1 RNase-free DNase, Promega). The RNAs were used to prepare the cDNAs using Superscript II reverse transcriptase (Invitrogen^TM^, Life Technologies, Carlsbad, CA, United States) and oligo (dT)15 primer. Quantitative real-time PCR reactions were performed using the SYBR green PCR master mix (Applied Biosystems, Foster City, CA, United States), in a StepOnePlus^TM^ real-time PCR system (Applied Biosystems, Foster City, CA, United States). The gene encoding pyruvate decarboxylase (PADG_00714) was selected for analysis. Constitutively expressed alpha tubulin and L34 were selected to normalize the samples (Borges et al., 2011). A cDNA aliquot from each sample diluted serially at 1:5 was mixed and used to generate a relative standard curve. The relative expression levels of selected genes were obtained using the standard curve method for relative quantification (Bookout et al., 2006). Statistical analysis employed Student’s *t*-test where *p*-values ≤ 0.05 were considered statistically significant.

### Mitochondrial Activity Assay

Mycelia, transition mycelia-to-yeast, and yeast cells of *P. brasiliensis* were grown in biological triplicates. The cells were harvested by centrifugation at 2,000 ×*g* for 5 min at 4°C and diluted in PBS buffer at the concentration of 10^6^ cells/mL. Then, the cells were stained with Rhodamine 123 (1.2 mM) (Sigma–Aldrich-Cas number 62669-70-9) according to the manufacturer’s protocol, followed by washing twice with 1× PBS. Stained cells were observed under a fluorescence microscope (AxioScope A1, Carl Zeiss), with the 546–512 nm filter. The imagens were acquired using the AxioVision Software (Carl Zeiss).

## Results

### Analysis of Proteins in Mycelium, Mycelium-to-Yeast Transition, and Yeast Cells

A large-scale proteome analysis was done for mycelium, mycelium-to-yeast transition, and yeast cells, in which an isobaric tag to proteins from each condition allowed relative quantification of the expressed proteins. A flow chart of the experimental steps is shown in Supplementary Figure S1.

**FIGURE 1:**
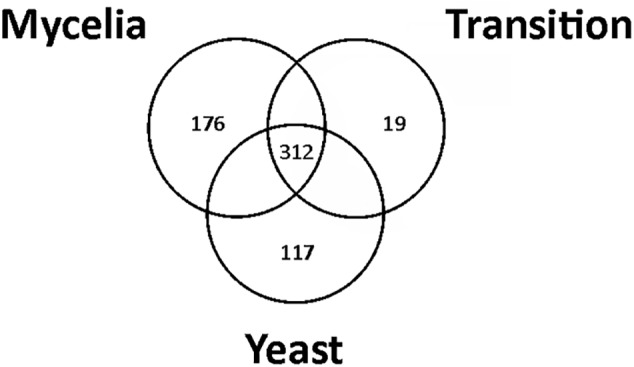
Venn diagram showing the overlap of common proteins identified in mycelium, transition mycelium-to-yeast, and yeast cells. The numbers indicate the total of proteins differentially expressed in mycelium, transition mycelium-to-yeast, and yeast cells.

Proteins with high FDR confidence and found in at least two replicates were selected for further analysis as shown in Supplementary Figure S2. One thousand and eight proteins were identified through mass spectrometry. In Supplementary Table S1, the protein accession number and description, the coverage of proteins, the number of peptides, unique peptides, groups of proteins, number of amino acids identified, protein molecular mass (kDa), and isoelectric point are presented. Statistical analysis, ANOVA (*p* ≤ 0.05), and Tukey’s test determined 312 differentially regulated *P. brasiliensis* proteins. Supplementary Table S2 summarizes all differentially expressed proteins from the three morphological phases; whereby the accession number and description of the protein, *p*-value determined by the ANOVA test, and average abundance obtained in the Tukey’s test are given. Furthermore, the identified proteins were clustered into functional categories by the functional catalog (Funcat 2.0) or KEGG terms and depicts the score of the proteins obtained from the MS Amanda 2.0 and *p*-value (≤0.05, ANOVA). Several proteins have been grouped as unclassified, since their biological function is still unknown. Most of these unclassified proteins are hypothetical (even after Blast2GO search), which may explain why no term can be assigned to most of these proteins (Supplementary Figure S3, panel A). Supplementary Figure S3, panel B depicted top-hit of species in the genus *Paracoccidioides* that were homologous to proteins found using Blast2GO. A diagram depicting the number of common proteins that have been identified in the mycelium, transition mycelium-to-yeast, and yeast cells protein extracts, with a total of 312 common proteins, is shown in Figure 1. Of these, 176 proteins were differentially expressed in mycelium, 19 in transition, and 117 in yeast cells, respectively (Figure 1).

### Metabolic Changes in the Mycelium

**FIGURE 2:**
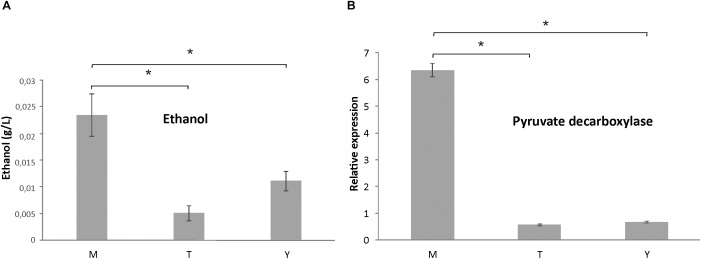
Ethanol measurements in protein extracts of mycelium, mycelium-to-yeast transition, and yeast cells, and analysis of the transcript encoding pyruvate decarboxylase by qRT-PCR. **(A)** Measurement of ethanol accumulation in *P. brasiliensis* morphological phases: M, mycelium; T, transition mycelium-to-yeast cells; Y, yeast cells. **(B)** Analysis of the abundance of the transcript encoding pyruvate decarboxylase. ^∗^Evidence statistical differences observed by Student’s *t*-test presenting *p-*value ≤ 0.05 considered significant.

At first, we compared the differentially expressed proteins in mycelium to the transition state and to yeast cells. In the metabolism category, mycelium presented up-regulation of proteins related to nucleotide/nucleoside/nucleobase, carbohydrate metabolism, phosphate metabolism, cell rescue, and secondary metabolism (Supplementary Table S3). We first focused on the category of genes encoding for proteins related to energy production. Mycelium presented a high number of up-regulated enzymes related to glycolysis and fermentation than the transition phase and yeast cells. For example, fructose-1,6-bisphosphate aldolase, class 2 (PADG_00852), hexokinase (PADG_07950), triosephosphate isomerase (PADG_06906), and 2,3-bisphosphoglycerate-independent phosphoglycerate mutase (PADG_05109) were up-regulated in mycelium. In respect to enzymes related to alcoholic fermentation, alcohol dehydrogenase ADH1 (PADG_11405) was induced in comparison to the other two developmental stages. This fact caught our attention since it had been previously described that *P. lutzii* presents a more anaerobic metabolism in yeast cells, when compared to mycelia (Felipe, 2005; Rezende et al., 2011). Therefore, we studied ethanol accumulation by comparing mycelium, transition of mycelium-to-yeast, and yeast phases, as shown in Figure 2, panel A. In agreement to the proteomic data, the mycelium showed a higher accumulation of ethanol than the other phases. In addition, analysis of the level of expression of alcohol dehydrogenase (PADG_11405) from proteomic analysis confirmed that the abundance of this enzyme was significantly higher in mycelium (Supplementary Table S3). Pyruvate decarboxylase (PADG_00714) is required to shunt pyruvate from glycolysis into the fermentative pathway of ethanol production by converting pyruvate into acetaldehyde. Therefore, we performed expression analyses on the respective transcript by real-time PCR (Figure 2B) demonstrating a six times higher expression in mycelium. This reinforces that in contrast to *P. lutzii* the mycelium from *P. brasiliensis* is dominated by a fermentative metabolism producing ethanol from pyruvate. This is also in line with a high activity of glycolysis since enzymes of the glycolytic pathway are induced in the mycelium (Supplementary Table S3).

**FIGURE 3:**
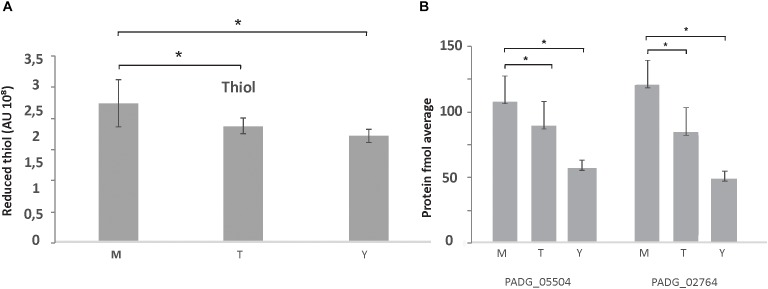
Thiol measurements in protein extracts of mycelium, mycelium-to-yeast transition, and yeast cells. **(A)** Production of thiol in *P. brasiliensis* morphological phases: M, mycelium; T, transition mycelium-to-yeast cells; Y, yeast cells. **(B)** Expression levels of the proteins for PADG_05504/Trx; PADG_02764/Trx, from proteomic analysis [fmol/μL (log2]). ^∗^Evidence statistical differences observed by Student’s *t*-test presenting *p-*value ≤ 0.05 considered significant.

Besides an increased fermentation capacity, proteins responsible for the maintenance of the intracellular redox state and protection against oxidative stress such as glutathione *S*-transferase Gst3 (PADG_03423), two Trx (PADG_02764, PADG_05504), mitochondrial peroxiredoxin PRX1 (PADG_03095), SOD [Cu–Zn] SOD1 (PADG_07418), and Fe–Mn family SOD SOD2 (PADG_01755) were up regulated in mycelium (Supplementary Table S3). In agreement with these proteomic data, evaluation of the enzymatic activity of Trx by measuring the thiol dosage revealed that mycelium produces more thiols than the other two morphological phases (Figure 3A). The thiol levels also correlated with the expression of Trx that were up-regulated in mycelium (Figure 3B).

We also detected major differences in the abundance of enzymes involved in cell wall biosynthesis and degradation. A chitinase class II (PADG_00994) showed an accumulation in mycelium (Supplementary Table S3). Additionally, other enzymes such as β-1,3-exoglucanase (PADG_03691), cell wall protein ECM33 precursor (PADG_04499), and β-1,3-glucosidase (PADG_02862) were more abundant in mycelium compared to the mycelium-to-yeast transition and yeast cells (Supplementary Table S3). A diagram depicting the processes potentially induced and repressed in the *P. brasiliensis* cell wall mycelia and yeast cells is shown in Supplementary Figure S4.

**FIGURE 4:**
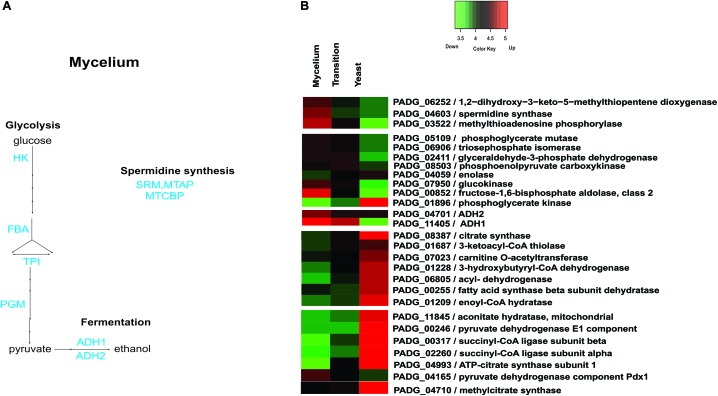
Schematic diagram of the metabolic processes differentially expressed in mycelia and heatmap of proteins in mycelium, mycelium-to-yeast transition, and yeast cells. **(A)** The figure summarizes the data obtained from proteomic analysis; enzymes are listed as follows: HK, hexokinase; FBA, fructose-1,6-bisphosphate aldolase; TPI, triosephosphate isomerase; PGM, phosphoglycerate mutase; ADH 1 and 2, alcohol dehydrogenase; MTAP, methylthioadenosine phosphorylase; SRM, spermidine synthase; MTCBP, 1,2-dihydroxy-3-keto-5-methylthiopentene dioxygenase. **(B)** The color scale shows the mean of abundance of proteins obtained that were differentially expressed by the ANOVA test (*p*-value ≤ 0.05). Functional categories were obtained by manual search in the annotation database Pedant (http://pedant.gsf.de/) on MIPS that provides a tool to search the Functional Categories (Funcat 2.0). Red represents significantly higher expression and green represents a significantly low level of expression.

Supplementary Table S4 depicts the down regulated proteins in mycelia compared to the other analyzed phases. Amino acid metabolism was down-regulated in mycelium compared to yeast cells, which is consistent with transcriptional data, in which *P. brasiliensis* revealed a predominance of up regulated transcripts related to the amino acid metabolism in yeast cells (Nunes et al., 2005). Furthermore, most enzymes of the tricarboxylic acid (TCA) cycle, electron transport, and oxidative phosphorylation were repressed in mycelium compared to mycelium-to-yeast transition and yeast cells (Supplementary Table S4). Most strikingly, all enzymes involved in beta-oxidation of fatty acids were down regulated (Supplementary Table S4). Those data reinforce the presence of a more anaerobic metabolism in the mycelium phase, compared to transition phase and yeast cells. These data demonstrate significant metabolic differences among members of the *Paracoccidioides* genus. While metabolism of mycelium from *P. brasiliensis* seems to follow a fermentative pathway, transcriptomic and proteomic analyses of the different morphological phases of *P. lutzzi* demonstrated a more anaerobic metabolism for yeast cells (Felipe, 2005; Rezende et al., 2011). The infective cells also induced three enzymes related to the production of the polyamine spermidine, which influences fungal morphogenesis (Kummasook et al., 2013). A heatmap and the related metabolic pathways that are induced and repressed in *P. brasiliensis* mycelia compared to mycelia-to-yeast transition and yeast cells are shown in Figure 4.

Interestingly, differences were also observed in proteins related to ribosome biogenesis, protein synthesis, protein folding, and stabilization (Supplementary Table S4). Here, these data indicate a decreased turnover of proteins in mycelium compared to mycelium-to-yeast transition and yeast cells.

### Metabolic Changes During the Mycelium-to-Yeast Transition Phase

Metabolic processes that predominate during the mycelium-to-yeast transition compared to mycelium and yeast cells are depicted in Supplementary Table S5. The accumulation of phosphoenolpyruvate carboxykinase (PADG_08503) suggests a shift of metabolism to gluconeogenesis at 22 h after entering the transition phase. This enzyme produces phosphoenolpyruvate, a precursor of glucose synthesis. Additionally, the exoantigen Gp43 (PADG_07615) accumulates during transition. However, despite significant changes on the transcript level (Nunes et al., 2005), overall only 19 proteins accumulated at the 22 h transition phase, which could be explained by the time required to translate a protein from a transcript.

Besides proteins strongly upregulated, the 22 h temperature shift period resulted in a reduction of several proteins that are involved in different metabolic processes (Supplementary Table S6). Proteins involved in amino acid metabolism were strongly down regulated. Similarly, the TCA, glycolysis, alcoholic fermentation, electron transport, and oxidative phosphorylation were repressed at this cellular stage, strongly suggesting a major repression of the overall cellular metabolism at this early time of transition.

### Metabolic Changes in the Established Yeast Phase

**FIGURE 5:**
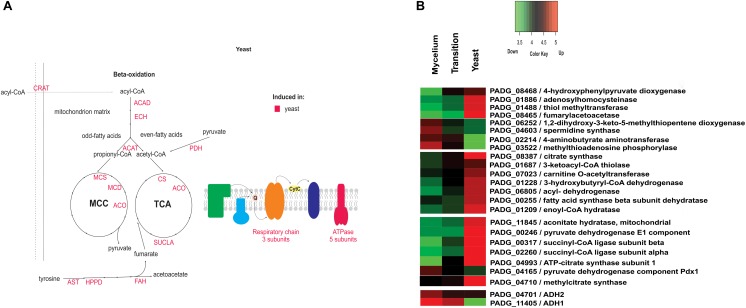
Schematic diagram of the metabolic processes differentially expressed in yeast cells and heatmap of proteins in mycelium, mycelium-to-yeast transition, and yeast cells. **(A)** The figure summarizes the data obtained from proteomic analysis; enzymes are listed as follows: Acad, acyl-CoA dehydrogenase; ECH, enoyl-CoA hydratase; ACAT, ketoacyl-CoA thiolase; MCS, methylcitrate synthase; MCD, methylcitrate dehydrogenase; ACO, aconitase; CS, citrate synthase; SUCLA, succinyl-CoA ligase; PDH, pyruvate dehydrogenase; ATPase, ATP synthase; ANT, aspartate aminotransferase; HPPD, hydroxyphenylpyruvate dioxygenase; FAH, fumarylacetoacetase; CRAT, carnitine acetyltransferase; SRM, spermidine synthase; MTAP, methylthioadenosine phosphorylase; MTCBP, 1,2-dihydroxy-3-keto-5-methylthiopentene dioxygenase. **(B)** The color scale shows the mean of abundance of differentially expressed proteins (*p*-value ≤ 0.05). Functional categories were obtained by manual search in the annotation database Pedant (http://pedant.gsf.de/) on MIPS that provides a tool to search the Functional Categories (Funcat 2.0). Red represents significantly higher expression and green represents a significantly low level of expression.

In yeast cells, several enzymes of amino acid metabolism were more abundant than in mycelium and during the transition phase (Supplementary Table S7 and Figure 5). In particular, the accumulation of 4-hydroxyphenylpyruvate dioxygenase (PADG_08468) an enzyme essential for tyrosine metabolism was observed in yeast cells, as depicted in Supplementary Table S7.

Furthermore, 24 ribosomal proteins showed an accumulation in yeast cells (Supplementary Table S7), which indicates an increased requirement for *de novo* protein biosynthesis in this morphological state. In terms of metabolic physiology, proteins related to the TCA cycle, such as pyruvate dehydrogenase (PADG_00246), ATP-citrate synthase (PADG_04993), succinyl-CoA ligase (PADG_02260 and PADG_00317), and aconitate hydratase (PADG_11845), were more abundant in yeast cells compared to transition phase and mycelium (Figure 5). Also, proteins involved in the electron transport chain and ATP synthase complex also were predominantly induced in yeast cells (PADG_07813, PADG_05402, PADG_08349, PADG_07042, PADG_02561), which agrees with an increased metabolite flux through the TCA cycle accompanied by aerobic respiration. Supplementary Figure S5 depicts activity assays from mycelia, transition phase, and yeast cells where it was possible to observe an increased aerobic respiration in yeast cells compared to mycelia and transition phase.

**FIGURE 6:**
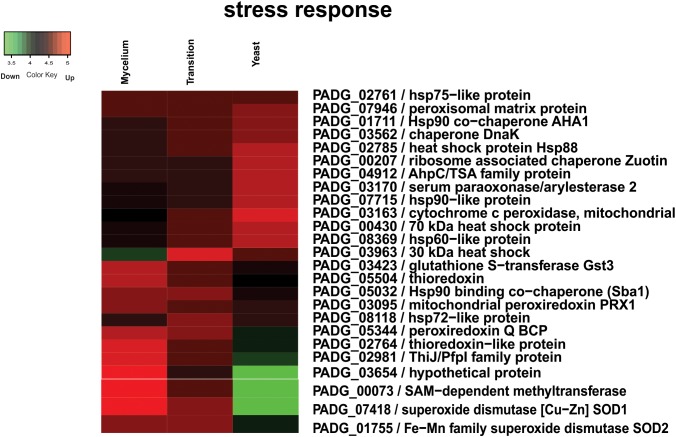
Heatmap of HSPs induced and repressed in yeast cells compared to mycelia and mycelia-to-yeast transition. The color scale shows the mean of abundance of differentially expressed proteins (*p*-value ≤ 0.05). Functional categories were obtained by manual search in the annotation database Pedant (http://pedant.gsf.de/) on MIPS that provides a tool to search the Functional Categories (Funcat 2.0). Red represents significantly higher expression and green represents a significantly low level of expression.

In fungi, regulation of production of HSPs is modulated in response to temperature. As the process of cell differentiation in *Paracoccidioides* spp. to the parasitic yeast phase is dependent on the temperature increase, HSPs are expected to increase during the morphological transition (Rappleye and Goldman, 2006). However, our data indicate that accumulation of HSPs does not immediately take place at 22 h after temperature shift but is slightly delayed with seven more abundant HSPs (PADG_01711, PADG_02785, PADG_07715, PADG_00430, PADG_08369 and PADG_02761) in the yeast phase. Figure 6 depicts a heatmap the HSPs induced and repressed in yeast cells compared to mycelia and mycelia-to-yeast transition. Besides HSPs, cytochrome c peroxidase, which is an important cell-rescue-related protein, accumulated in yeast cells compared to the other phases (Figure 6).

Evaluation of proteins at the yeast phase revealed down regulation of alcohol dehydrogenase ADH1 (PADG_11405) (Supplementary Table S8). Of special note six enzymes of the glycolysis, including hexokinase, were down regulated in yeast cells compared to mycelia and mycelia-to-yeast transition.

We also observed a dramatic change in the composition of cell wall-related enzymes. The chitinase class II (PADG_00994), β-1,3-glucosidase (PADG_02862), and β-1,3-exoglucanase (PADG_03691) showed a significant decrease (Supplementary Table S8 and Supplementary Figure S4). Especially the decrease in the amount of chitinase correlates to the high amount of chitin found in yeast cells (San-Blas, 1982).

## Discussion

Proteomic analysis of the three morphological phases of *P. brasiliensis* revealed unexpected data related to carbon source utilization and energy production. While yeast cells of *P. lutzii* use glycolysis and fermentation as main energy production pathways, *P. brasiliensis* yeast cells rely on the aerobic beta-oxidation and the TCA cycle for ATP production. This observation is sustained by the accumulation of enzymes such as acyl-CoA dehydrogenase (EC:1.3.8.1), enoyl-CoA hydratase (EC:4.2.1.17), aconitate hydratase (EC:4.2.1.3), thiolase (EC:2.3.1.16), citrate synthase (EC:2.3.3.1), succinyl-CoA transferase (EC:2.8.3.5), and others that were highly present in yeast cells. In addition, eight proteins of oxidative phosphorylation were induced in the yeast form. Furthermore, besides an accumulation of enzymes involved in beta-oxidation of fatty acids, an accumulation of methylcitrate synthase and methylcitrate dehydratase was observed. These enzymes play an essential role in the methylcitrate cycle and convert propionyl-CoA into pyruvate. As propionyl-CoA can derive from the beta-oxidation of odd-chain fatty acids and from degradation of amino acids, this metabolic pathway may be essential for increasing the nutritional status of yeast cells by simultaneously preventing the accumulation of toxic propionyl-CoA that may derive the degradation of amino acids (Brock and Buckel, 2004).

In addition, proteins related to the TCA were more abundant in yeast cells compared to transition phase and mycelium and enzymes of glycolysis were down regulated. These data strongly support the observed metabolic preference for the TCA cycle and respiration and are in agreement with similar observations in *Talaromyces marneffei* (Pasricha et al., 2017). This is also in accordance with biochemical data published for *Paracoccidioides* sp. in which Medoff et al. (1987) described increasing concentrations of cytochrome components and resumption of the normal respiration in yeast cells 5 days after transition. Interestingly, our data indicate that metabolism appears not to be directly dependent on glucose utilization, as all enzymes involved in beta-oxidation of fatty acids were highly abundant in yeast cells compared to the mycelia-to-yeast transition and mycelia. In agreement, during *in vivo* infection of lungs by *P. brasiliensis*, enzymes related to lipid degradation were up-regulated in yeast cells (Lacerda Pigosso et al., 2017).

In agreement with a limited glucose supply during the parasitic phase, the metabolism of amino acids seems important for adaptation to the host environment. This may explain the increased abundance of proteins involved in amino acid metabolism in yeast cells of *P. brasiliensis* (Costa et al., 2007; Zhang and Rubin, 2013). The highly abundant protein 4-HPPD (EC:1.13.11.27) is related to tyrosine degradation and had been described as a new potential drug target, since the use of the specific 4-HPPD inhibitor NTBC [2-(2-nitro-4trifluoromethyl-benzoyl)-cyclohexane-1,3-dione] prevents the dimorphic transition in a dose-dependent manner (Nunes et al., 2005). Studies on *T. marneffei* yeast cells revealed a lower consumption rate of glucose in yeast cells compared to hyphae accompanied by the utilization of several amino acids that are likely to undergo deamination and fuel the TCA cycle. According to the authors, this shift to a more efficient energy metabolism reduces the requirement for exogenous glucose in *T. marneffei*, allowing for an optimization of nutrient utilization in the limited environment of macrophages (Pasricha et al., 2017).

We also observed an accumulation of the enzyme adenosylhomocysteinase (EC:3.3.1.1) in yeast cells of *P. brasiliensis*. This enzyme degrades *S*-adenosylhomocysteine, which is a strong inhibitor of *S*-adenosyl methionine-dependent methyltransferases, which is essential for the synthesis of the phospholipid phosphatidylcholine, preferentially found in yeast cells (Manocha, 1980; Malanovic et al., 2008).

When we analyzed the transition phase it was possible to note an accumulation of phosphoenolpyruvate carboxykinase. In *P. brasiliensis*, the enzyme phosphoenolpyruvate carboxykinase has been described as relevant for metabolic adaptation within macrophages (Derengowski et al., 2008). Moreover, proteins involved in amino acid metabolism, TCA, glycolysis, alcoholic fermentation, electron transport, and oxidative phosphorylation were down regulated. According to Medoff et al. (1987), immediately after the temperature shift, the metabolism of *Paracoccidioides* is characterized by partial or complete uncoupling of oxidative phosphorylation and decline in ATP levels. Subsequently, there is a dormant period of 4–6 days that is characterized by absent or low rates of respiration and inhibition of protein synthesis (Medoff et al., 1987). Our proteomic data showing a high number of repressed proteins corroborate with the description of the metabolic changes described above.

The alcoholic fermentation of glucose was up-regulated in mycelium. This is supported by the accumulation of glycolytic enzymes such as the glycolysis-specific enzyme hexokinase (EC:2.7.1.1), fructose 1,6-biphosphate aldolase (EC:4.1.2.13), triose phosphate isomerase (EC:5.3.1.1), and phosphoglycerate mutase (EC:5.3.1.1), as well as alcohol dehydrogenase ADH1 (EC:1.1.1.1). Although a dominant fermentative metabolism of *P. brasiliensis* mycelium was not expected, this data agree with the dimorphic fungus *T. marneffei* in which hyphae also predominantly exhibit a fermentative metabolism with the production of ethanol and a minimum flow of pyruvate through the citric acid cycle.

While our data strongly suggest that *P. brasiliensis* presents a more anaerobic metabolism in the mycelium compared to mycelia-to-yeast transition phase and yeast cells, previous transcriptional studies with *P. brasiliensis* undergoing mycelia-to-yeast transition using a biochip detected an induction of transcripts encoding alcohol dehydrogenase I and pyruvate decarboxylase during the dimorphic transition from mycelia-to-yeast cells. However, the latter data were obtained at last 120 h after dimorphic transition, which could explain the difference in the transcript levels compared to the protocol used here (Nunes et al., 2005).

As observed in our results, higher expression of pyruvate decarboxylase in the form of mycelium confirmed that *P. brasiliensis* mycelial form has an anaerobic metabolism with ethanol production, which can be produced from pyruvate, acetate, and acetaldehyde. Comparative proteomic studies among isolates *P. lutzii*, *P. americana*, and *P. restrepiensis* in a nutrient-rich media revealed a high level of ethanol production in *P. lutzii* compared to the other isolates (Pigosso et al., 2013).

Besides the carbon flux through metabolic pathways, remodeling of the cell wall during the transition from mycelium-to-yeast cells is vital. As with other fungi, the cell wall of *P. brasiliensis* is a network of glycoproteins and polysaccharides that are responsible to protect the fungal cell from environmental stress (De Groot et al., 2005) and confers virulence to the fungus. Compared to mycelium, an increase in chitin levels in yeast phase was detected in *P. brasiliensis*, which defines the cell wall thickness (Kanetsuna et al., 1969). Furthermore, the chitin degrading chitinase CTS2 was accumulated in mycelium and decreased in the yeast phase. Moreover, glucans account for approximately 40% of the cell wall in mycelium and yeast cells of *P. brasiliensis* (Kanetsuna et al., 1969). Alpha glucan predominates in the cell wall glucan content of the yeast form, whereas β-glucan predominates in the mycelia (Kanetsuna and Carbonell, 1970; San-Blas and San-Blas, 1977). An α-glucan layer is essential for avoiding dectin-1-mediated phagocytosis of yeast cells by macrophages, by masking the β-1,3-glucan layer, as shown for *Histoplasma capsulatum* chemotype II cells (Edwards et al., 2011). In this way, variations in cell wall glucans may play a key role in the fungus dimorphism and, thus, its pathogenesis.

For a remodeling of the glucan structure, β-(1,3)-glucanase plays a key role in morphogenetic processes by hydrolyzing the β-glucan chain, which is largely predominant in mycelial phase (Adams, 2004). In agreement with an alteration of the glucan structure, a β-(1,3)-exoglucanase was up regulated in mycelium and transition of mycelium-to-yeast cells. Furthermore, other cell wall-related proteins such as Ecm33 were differentially produced in mycelia. Ecm33 is a GPI-linked cell wall protein that plays an important role for cell wall integrity and architecture in *C. albicans* (Martinez-Lopez et al., 2004, 2006). In *P. lutzii*, this protein occurs in the mycelium cell wall, in agreement to the data here presented (Araújo et al., 2017).

In respect to cell-rescue, cytochrome c peroxidase was increased in yeast cells compared to the other phases. This enzyme is involved in *P. brasiliensis* in the response to oxidative and nitrosative stresses and mutants with low expression of the gene were more sensitive to nitrosative stress (Parente-Rocha et al., 2015). Furthermore, antisense knockdown mutants of *P. brasiliensis* revealed a decreased survival inside macrophages and *in vivo* infection (Parente-Rocha et al., 2015). The higher expression in yeast cells added of the previous data corroborate the protein to be designated as a virulence factor. Additional virulence determinants were upregulated mainly in the mycelium-to-yeast transition phase and yeast cells, with a special importance of HSPs, and proteins associated to cell adhesion.

As the process of cell differentiation in *Paracoccidioides* spp. to the parasitic phase is related to increase of the temperature, the HSPs are expected to accumulate during the morphological transition (Rappleye and Goldman, 2006). The HSPs are activated in an attempt to suppress ROS production, allowing the adaptation of the fungus to the temperature (Burnie et al., 2006). Proteins related with maintenance of the intracellular redox and protection against oxidative stress were also identified in this comparison, such as SODs, Cu/Zn-containing (SOD1), and Fe/Mn-containing (SOD2), glutathione *S*-transferase, Trx, peroxiredoxin PRX, were up-regulated in the mycelia phase. Analysis of gene expression between phylogenetic lineages of *Paracoccidioides* revealed the induction of SOD1 was higher for S1, PS2, and *P. lutzii* isolates in mycelia, suggesting that the gene may exhibit phase-dependent expression and possibly be necessary for defense against ROS produced endogenously (Tamayo et al., 2016). Those virulence determinants can be assumed to be relevant for the fungus to establish an infection. The data obtained here reinforce that during the morphological transition, *P. brasiliensis* undergoes a metabolic reorganization for adapting to the increased temperature and nutritional environment in the host.

Members of the *Paracoccidioides* complex can infect diverse host niches, an aspect that may be correlated to metabolic plasticity to cope with diverse carbon source availability. As have been demonstrated by our group, following exposure to the liver, which is presumably a glucose-rich environment, *P. lutzii* accumulates transcripts of genes associated with glycolysis along with alcohol fermentation (Costa et al., 2007). Additionally, *P. brasiliensis* accumulates enzymes of beta-oxidation during lung infection (Lacerda Pigosso et al., 2017). If this metabolic plasticity correlates with fungi adaptation to host niches remains to be elucidated. To our knowledge there are no descriptions comparing the metabolism of *P. lutzii* and *P. brasiliensis* at different host niches. Studies with this focus will be performed by our group.

## Author Contributions

CS and DA conceived and designed the experiments. DA, IP, ASJ, WF, LA, and LB performed the experiments. DA, MP, WF, LB, AB, CR, and CS analyzed and/or interpreted the data. CS, MS, WF, and CR contributed to reagents and materials. DA, MP, LB, AB, MB, and CS analyzed the data and wrote the manuscript.

## Conflict of Interest Statement

The authors declare that the research was conducted in the absence of any commercial or financial relationships that could be construed as a potential conflict of interest.

## Footnotes

1 http://www.r-project.org/

2 https://www.blast2go.com/

3 http://pedant.gsf.de/genomes.jsp?prefix=P&category=fungal

4 http://pedant.gsf.de/

## Supplementary Material

FIGURE S1 Schematic overview of performed experiments. Nine cultures were prepared with *P. brasiliensis*, grown as mycelium, mycelium-to-yeast, and yeast cells. After 72 h of growth for mycelium and yeast and 22 h for transition, the cultures were collected and the proteins extracted and digested in peptides. The three-condition samples (mycelium, transition, and yeast) from each replicate were labeled with iTRAQ (114, 116, and 115 tag).

FIGURE S2 Flowchart of the filters applied to the identified proteins during mycelium, mycelium-to-yeast transition, and yeast cells. It was considered those proteins with high or medium FDR confidence and those that were found in at least two replicates.

FIGURE S3 Proteins identified in *P. brasiliensis* that were annotated by Blast2GO. **(A)** Number of sequences analyzed and that had annotation. **(B)** Top-hit of species that were homologous to proteins found using Blast2GO.

FIGURE S4 Schematic diagram of the metabolic processes in cell wall differentially expressed in mycelia and yeast cells. The figure summarizes the data obtained from proteomic analysis; enzymes are listed as follows: CTS2, chitinase; ECM33; β-1,3-exoglucanase; 1,3-β-glucosidase; β-1,6 glucan synthase (Knh1); M, mycelium; T, transition from mycelium to yeast cells; Y, yeast cells.

FIGURE S5 Mitochondrial activity assay in mycelia, transition mycelia-to-yeast, and yeast cells. The images were acquired to mycelia, transition mycelia-to-yeast, and yeast cells (magnification 400×) in the Microscope Axio Scope A1 (Zeiss), using the AxioVision Software (Carl Zeiss).
