# Supplementary figures and images for: Metabolic Peculiarities of Paracoccidioides brasiliensis Dimorphism as Demonstrated by iTRAQ Labeling Proteomics
Source: Front Microbiol. 2019 Mar 20;10:555. doi: 10.3389/fmicb.2019.00555 (PMC6436475; doi:10.3389/fmicb.2019.00555)

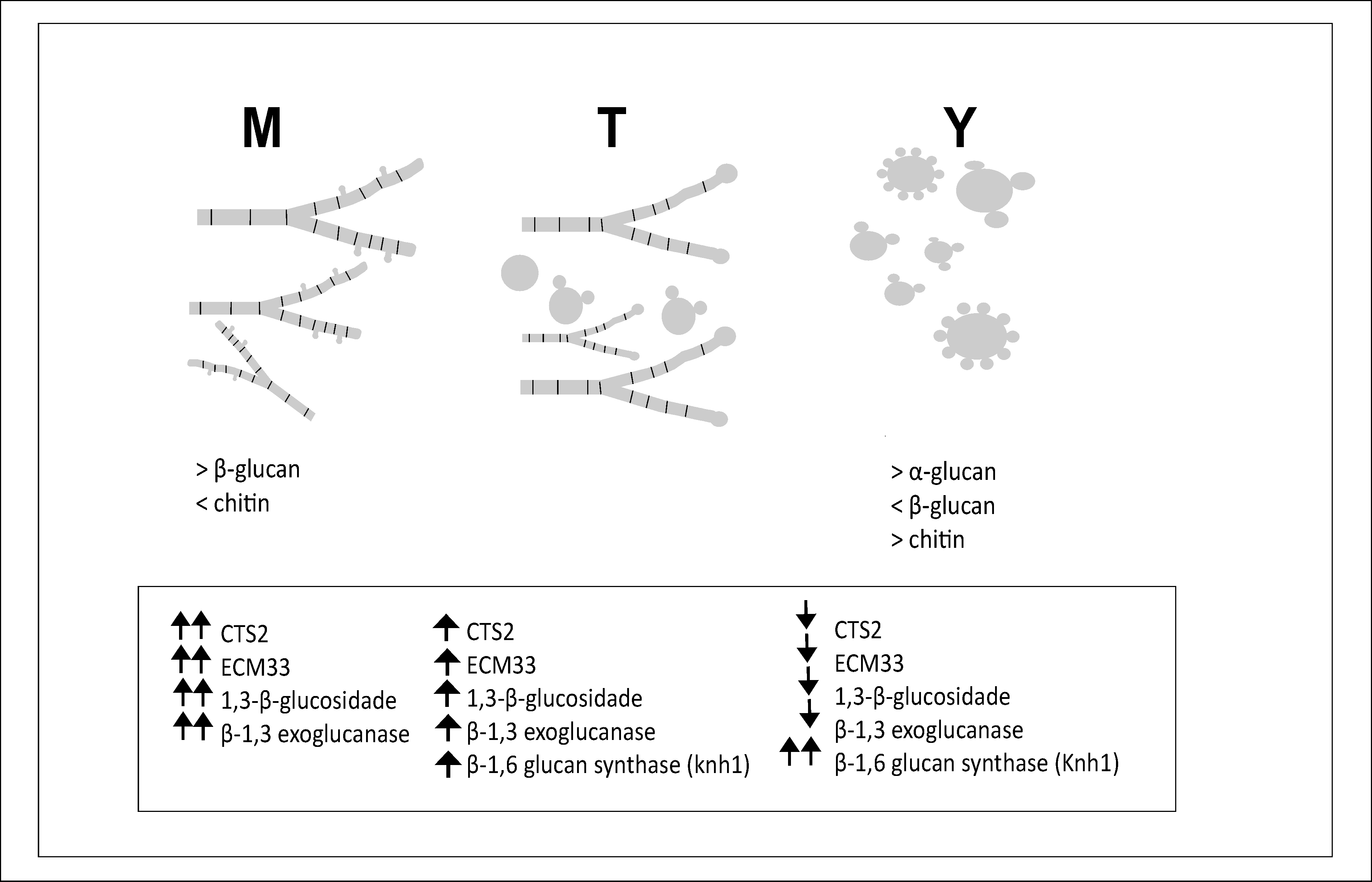

Supplement: FIGURE S4 — Schematic diagram of the metabolic processes in cell wall differentially expressed in mycelia and yeast cells. The figure summarizes the data obtained from proteomic analysis; enzymes are listed as follows: CTS2, chitinase; ECM33; β-1,3-exoglucanase; 1,3-β-glucosidase; β-1,6 glucan synthase (Knh1); M, mycelium; T, transition from mycelium to yeast cells; Y, yeast cells. [file Image_4.TIF]
